# Are Multiple Mitochondrial Related Signalling Pathways Involved in Carotid Body Oxygen Sensing?

**DOI:** 10.3389/fphys.2022.908617

**Published:** 2022-05-31

**Authors:** Andrew P. Holmes, Agnieszka Swiderska, Demitris Nathanael, Hayyaf S. Aldossary, Clare J. Ray, Andrew M. Coney, Prem Kumar

**Affiliations:** ^1^ School of Biomedical Sciences, Institute of Clinical Sciences, College of Medical and Dental Sciences, University of Birmingham, Birmingham, United Kingdom; ^2^ Unit of Cardiac Physiology, Division of Cardiovascular Sciences, Faculty of Biology, Medicine and Health, University of Manchester, Manchester, United Kingdom; ^3^ College of Medicine, Basic Medical Sciences, King Saud bin Abdulaziz University for Health Sciences, Riyadh, Saudi Arabia

**Keywords:** carotid body, hypoxia, mitochondria, succinate, arterial chemoreceptor, O_2_ sensor, metabolism, mitochondrial inhibitors

## Abstract

It is generally acknowledged that the carotid body (CB) type I cell mitochondria are unique, being inhibited by relatively small falls in P_a_O_2_ well above those known to inhibit electron transport in other cell types. This feature is suggested to allow for the CB to function as an acute O_2_ sensor, being stimulated and activating systemic protective reflexes before the metabolism of other cells becomes compromised. What is less clear is precisely how a fall in mitochondrial activity links to type I cell depolarisation, a process that is required for initiation of the chemotransduction cascade and post-synaptic action potential generation. Multiple mitochondrial/metabolic signalling mechanisms have been proposed including local generation of mitochondrial reactive oxygen species (mitoROS), a change in mitochondrial/cellular redox status, a fall in MgATP and an increase in lactate. Although each mechanism is based on compelling experimental evidence, they are all not without question. The current review aims to explore the importance of each of these signalling pathways in mediating the overall CB response to hypoxia. We suggest that there is unlikely to be a single mechanism, but instead multiple mitochondrial related signalling pathways are recruited at different P_a_O_2_s during hypoxia. Furthermore, it still remains to be determined if mitochondrial signalling acts independently or in partnership with extra-mitochondrial O_2_-sensors.

## Introduction—A Role for Mitochondria in Carotid Body O_2_ Sensing

The carotid body (CB) type I or glomus cell has an extraordinary ability to be stimulated by relatively small falls in P_a_O_2_, well above those that start to impact on the metabolism of other cell types (Kumar and Prabhakar, 2012). This allows the CB to act as a protective peripheral chemoreceptor that detects and responds to arterial hypoxia, leading to downstream reflex activation (Kumar, 2009). Of the proposed O_2_ sensors within the CB, including the mitochondria, haem-oxygenase 2, NADPH-oxidase and O_2_-dependent K^+^ ion channels, only the mitochondria have been identified as having a unique phenotype in comparison with other O_2_-insensitive cell types (Holmes et al., 2018a) and it has become increasingly apparent that the type I cell mitochondria have an important role in CB O_2_ sensing.

O_2_ binding within mitochondria takes place within the CuB/haem a_3_ (cytochrome a_3_) binuclear centre of cytochrome c oxidase (complex IV). Multiple groups have shown that the CB mitochondrial electron transport chain is inhibited as the O_2_ drops below an unusually and uniquely high PO_2_ threshold (Duchen and Biscoe, 1992; Buckler and Turner, 2013). The K_m_ of cytochrome c oxidase for O_2_ in the CB is remarkably high (Buckler and Turner, 2013) and cytochrome a_3_ reduction status is excessive over the physiological range of PO_2_s known to stimulate the type I cell (Mills and Jobsis, 1970; Mills and Jobsis, 1972). Emerging evidence also suggests that the CB type I cell has a unique mitochondrial gene expression signature, characterised by relatively high mRNA levels of *Ndufa4l2*, *Cox4i2*, and *Cox8b* (Zhou et al., 2016; Gao et al., 2017), with the latter two coding for important cytochrome c oxidase proteins. These genes are regulated in part by HIF2α, a critical transcription factor that underpins CB development, adult function, and plasticity in response to chronic hypoxia (Hodson et al., 2016; Macias et al., 2018; Moreno-Dominguez et al., 2020). Conditional deletion of *Cox4i2* in tyrosine hydroxylase (TH) -positive CB type I cells diminishes both the type I cell and ventilatory response to hypoxia in the mouse (Moreno-Dominguez et al., 2020). Indeed, competitive inhibition of O_2_ binding into cytochrome c oxidase using NO donors mimics hypoxia by causing intense chemoafferent stimulation when delivered at high concentrations, and augmented chemosensitivity at more moderate levels (Iturriaga et al., 2000; Mosqueira and Iturriaga, 2002; Holmes et al., 2016). Furthermore, saturating concentrations of exogenous NaCN, a cytochrome c oxidase inhibitor, act to abolish any further K^+^ channel inhibition induced by hypoxia (Wyatt and Buckler, 2004). Collectively, these findings point to impaired binding of O_2_ into cytochrome c oxidase and a subsequent run-down in mitochondrial activity as key events of hypoxia sensing in the CB.

**FIGURE 1:**
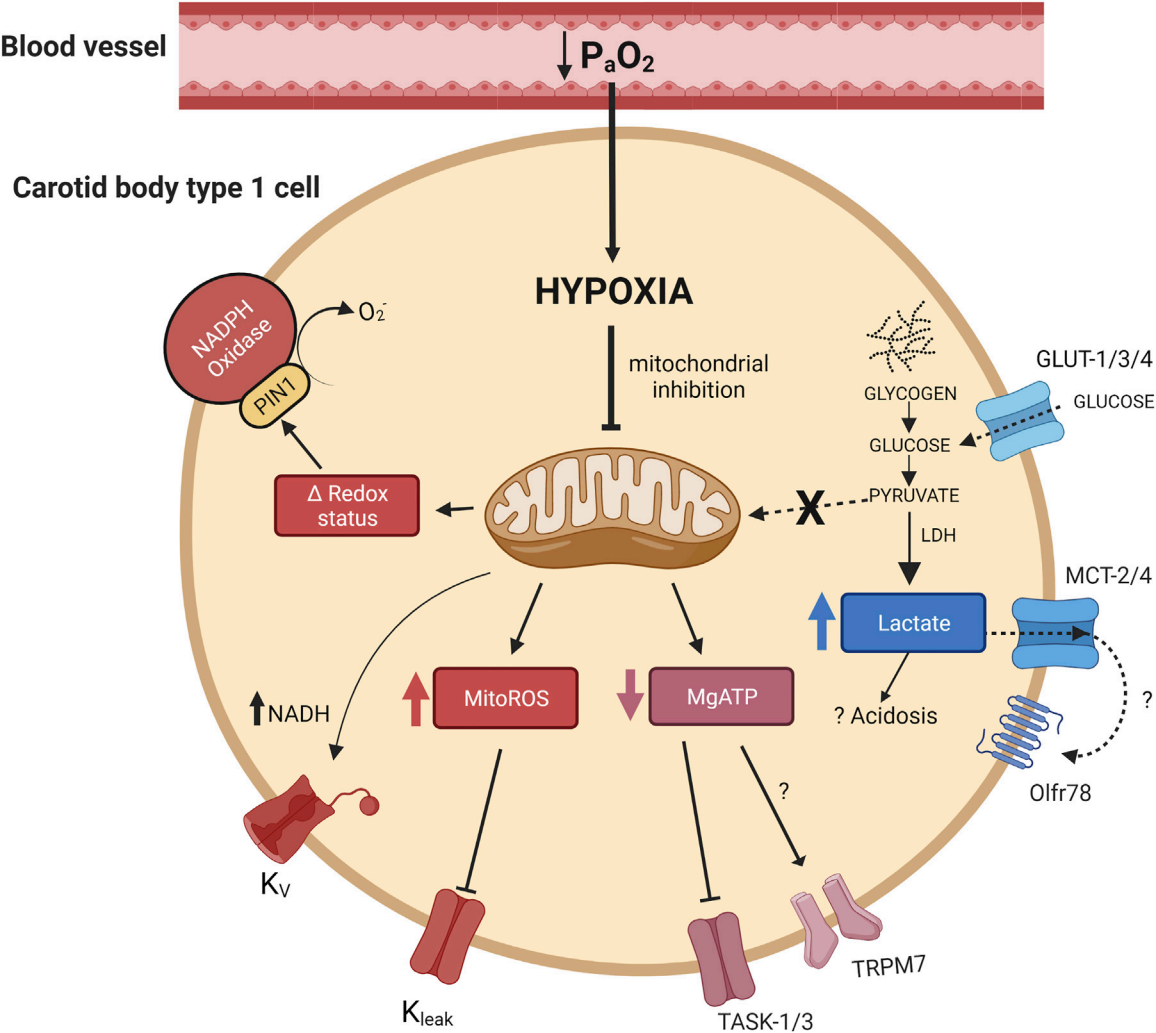
Activation of multiple mitochondrial related signalling pathways in the carotid body type I cell during hypoxia. Hypoxia leads to mitochondrial inhibition which is proposed to cause: 1) an elevation in mitochondrial reactive oxygen species (MitoROS), 2) a shift in mitochondrial/cellular redox status (Δ redox status), 3) a fall in MgATP and 4) a rise in lactate. Some of these pathways have been shown to directly modify ion channel activity and cause cellular depolarisation. NADH has also been suggested to modify voltage dependent potassium channels. Arrowheads indicate promotion, flatheads indicate inhibition. PIN1, peptidyl-prolyl cis/trans isomerase; Olfr78, olfactory receptor 78; TASK, TWIK-related acid-sensitive potassium channel; TRPM7, transient receptor potential channel M7; MCT-2, monocarboxylate transporter-2; MCT-4, monocarboxylate transporter-4; LDH, lactate dehydrogenase; K_v_, voltage dependent potassium channel(s), K_leak_, potassium leak channel(s).

An important consideration is how a fall in mitochondrial activity might then lead to activation of the CB chemotransduction cascade? This has been an important research focus over recent years and a better understanding of these specific mechanisms could offer new strategies to manipulate CB function in health and disease (Chang, 2017). A number of mitochondrial/metabolic signalling pathways have been proposed including an elevation in mitochondrial reactive oxygen species (mitoROS) generation (Fernandez-Aguera et al., 2015; Arias-Mayenco et al., 2018; Swiderska et al., 2021), an alteration in nicotinamide adenine dinucleotide (NAD^+^) and flavoprotein (Fp) redox status (Bernardini et al., 2020), a fall in MgATP (Varas et al., 2007) and an increase in lactate production (Chang et al., 2015) (Figure 1). Although there is compelling evidence for each of these processes, they are all not without question. The aim of this review is to explore the relative importance of each pathway and to consider the possibility that at least two or more of these may be needed to co-operate to produce full CB chemoexcitation during hypoxia.

## Are Mitochondrial ROS and Changes in NAD^+^/Fp Redox Status Required for CB Activation in Hypoxia?

The mitochondria are the main generators of ROS in most mammalian cells, with complex I and III considered to be the primary sources (Murphy, 2009). Using redox-sensitive green fluorescent protein (roGFP) targeted to selective mitochondrial compartments, Fernández-Agüera and colleagues demonstrated that ROS were elevated during hypoxia in the mitochondrial intermembrane space (IMS) (Fernandez-Aguera et al., 2015). Importantly, the authors showed that the increase in mitochondrial IMS ROS occurred over a similar physiological PO_2_ range known to cause type I cell excitation (Arias-Mayenco et al., 2018). This rise was not apparent in mouse type I cells that had deletion of the *Ndufs2* gene, suggesting that production of ROS in hypoxia was solely at complex I. In contrast, it was found that mitochondrial matrix ROS was decreased upon hypoxic exposure in both wild-type (WT) and *Ndufs2* deficient type I cells. Consistent with the idea that complex I derived ROS is an important effector signalling molecule, mice with conditional or inducible deletion of *Ndufs2 (Ndufs2-KO)* displayed a significantly attenuated rise in respiratory frequency when challenged by acute exposure to 10% environmental O_2_ (Fernandez-Aguera et al., 2015; Arias-Mayenco et al., 2018). Type I cells isolated from these mice also exhibited strong depression of neurosecretory and Ca^2+^ responses to acute hypoxia. These cells did, however, respond to hypercapnia, confirming functionality and selective depletion in O_2_ sensing capabilities. The *Ndufs2* gene appears to be of particular importance as genetic deletion of *Mt-nd6*, (the mitochondrial encoded subunit six of the NADH dehydrogenase complex), did not impair CB or ventilatory responses to hypoxia (Gomez-Nino et al., 2018). Intracellular delivery of exogenous H_2_O_2_ has been shown to increase input resistance, indicative of an inhibitory action on leak K^+^ channels (Fernandez-Aguera et al., 2015). The specific identity of such channels is still to be elucidated and the effect of ROS on voltage-dependent K^+^ channels remains to be further studied. A key future experiment will be to examine whether mitochondrial derived ROS can cause complete or partial block of TASK and/or BK_Ca_, two key K^+^ channels thought to be involved in rodent and human CB chemostimulation (Wyatt and Peers, 1995; Peers and Wyatt, 2007; Mkrtchian et al., 2012; Buckler, 2015). It will also be interesting to examine the distance between mitochondria and effector ion channels, which could, potentially, form discreet and tightly controlled redox-sensitive signalling microdomains.

If mitochondrial complex I derived ROS are essential, then it would be expected that pharmacological targeting of complex I should abolish or severely reduce CB responses to hypoxia. In CB slices isolated from rats, application of rotenone, a mitochondrial complex I inhibitor, has been shown to inhibit secretory responses to hypoxia in a concentration dependent manner (Ortega-Saenz et al., 2003). Similar results have been observed in terms of rotenone being able to abolish any further depression of background K^+^ currents caused by hypoxia (Wyatt and Buckler, 2004). However, in these latter experiments, a similar attenuation of hypoxic sensing was also observed by NaCN and FCCP (a mitochondrial uncoupler), which act independently of complex I. Furthermore, robust rises in Ca^2+^ induced by hypoxia have been observed in the presence of rotenone, with the concurrent application of ascorbate and TMPD, a cocktail suggested to deliver electrons directly to cytochrome c and subsequently maintain electron flow through to cytochrome c oxidase (Wyatt and Buckler, 2004). A similar restoration of the responses to NO donors (thought to excite the CB by preventing O_2_ binding into cytochrome c oxidase) can be achieved using ascorbate and TMPD following blockade of upstream mitochondrial complexes (Mosqueira and Iturriaga, 2002). Interestingly, it has also been reported by many groups that the CB can still be excited under conditions of complete anoxia, when the generation of ROS would theoretically be zero (Buckler and Turner, 2013; Peng et al., 2019). Whether or not complete removal of O_2_ from an *ex vivo* experimental set up can be achieved is debatable. Nevertheless, these experiments do point towards the existence of a substantial signalling pathway activated upon inhibition of cytochrome c oxidase that is independent of complex I and ROS generation.

**FIGURE 2:**
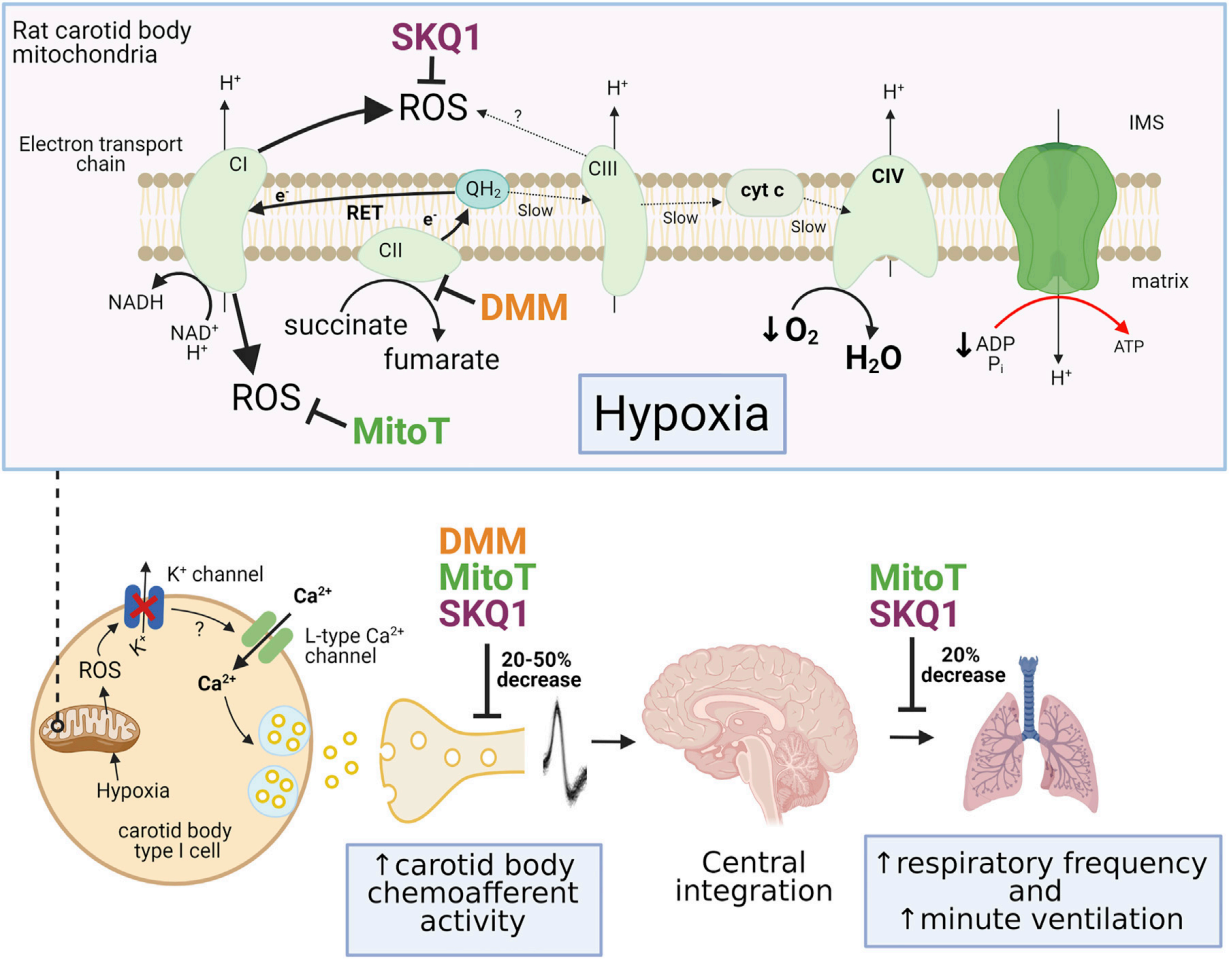
Summary of the effects of pharmacological inhibition of mitochondrial succinate metabolism and antioxidant treatment on carotid body and ventilatory responses to hypoxia. Top panel illustrates the mechanism of mitochondrial reactive oxygen species (ROS) generation through enhanced succinate metabolism at complex II (CII) and reverse electron transport (RET) during hypoxia. The sites of action of dimethyl malonate (DMM; complex II inhibitor), 10-(6′-plastoquinonyl) decyltriphenylphosphonium (SKQ1) and MitoTEMPO (MitoT) are also shown. Lower panel shows the inhibitory effect of these pharmacological agents on the rises in carotid body chemoafferent discharge frequency, respiratory frequency and minute ventilation during hypoxia. IMS, intermembrane space; cyt c, cytochrome c. Adapted from (López-Barneo and Ortega-Sáenz, 2022), (Swiderska et al., 2021) and (Chouchani et al., 2014).

To probe the importance of mitochondrial ROS further, we have recently measured rat CB chemoafferent activity and ventilation in the presence of the mitochondrial antioxidants, MitoTEMPO and SKQ1 (Swiderska et al., 2021). Both agents caused a moderate decrease (20%–50%) of the chemoafferent response to hypoxia, although a significant component remained intact (Figure 2). MitoTEMPO and SKQ1 produced a similar attenuation of the rise in respiratory frequency upon exposure to 10% environmental O_2_. SKQ1, a mitochondrial antioxidant targeted to the IMS, also led to an approximately 20% decrease in the hypoxic ventilatory response, whilst the response to hypercapnia was completely preserved. We cannot rule out the possibility that some mitoROS were not completely removed by these agents and there was also the potential for non-selective actions, e.g. on the post-synaptic afferent fibres and brain stem neurones. However, these data are more consistent with the idea that mitoROS are only partially responsible for eliciting CB stimulation during hypoxia.

The exact mechanism of mitochondrial ROS production is another important consideration. The idea that complex I ROS can be generated in particularly large amounts following excessive succinate metabolism at complex II and reverse electron transport (RET) gained significant attention due to its association with mediating cardiac myocyte cell death following ischemia-reperfusion (Chouchani et al., 2014). The strong link between succinate dehydrogenase (SDH; complex II) mutations and paraganglioma (tumour of the CB) does suggest that succinate metabolism has a heightened importance in the CB (Favier et al., 2015; Kluckova and Tennant, 2018). Interestingly, the CB type I cell does appear to contain an unusually high concentration of succinate in comparison with brain and adrenal medulla (Fernandez-Aguera et al., 2015). Conditional homozygous deletion of the *Sdhd* gene leads to a powerful attenuation of the Ca^2+^ response to hypoxia in isolated type I cells (Gao et al., 2017). However, these cells also exhibited similar depressions in response to high K^+^, possibly indicative of a more general impairment of CB function. In contrast, less severe heterozygous deletion of *Sdhd* was found to augment basal neurosecretion and have no impact on the hypoxic sensitivity (Piruat et al., 2004).

In our own experiments, exogenous application of the cell permeable form of succinate (diethyl succinate, DESucc) did lead to chemoafferent stimulation in the isolated rat CB, but this measured only approximately 10–30% of that induced by a subsequent severe hypoxic stimulus (Swiderska et al., 2021). Addition of a high concentration of dimethyl malonate (DMM; a competitive inhibitor of SDH) also produced a significant rise in basal chemoafferent frequency and caused partial (20%–50%) reduction of the activity in hypoxia. This was consistent with the level of hypoxic inhibition we observed in the presence of MitoTEMPO and SKQ1. It could be argued that DMM failed to completely block SDH activity in these experiments, although this concentration was sufficient to completely prevent the rise in chemoafferent activity induced by exogenous DESucc. Other groups have also seen partial attenuation of hypoxic neurosecretion in the presence of DMM, with a significant proportion of the response still being preserved (Arias-Mayenco et al., 2018). DMM also caused significant, but not complete, attenuation of IMS ROS generation evoked by hypoxia. Taken together, these data do suggest that succinate metabolism is important for ROS generation and CB stimulation during hypoxia but is not the sole mechanism. Furthermore, it is possible that there is an alternative mode of IMS mitochondrial ROS production that is independent of succinate metabolism. An intriguing mechanism is the potential generation of ROS from mitochondrial complex III. The functional importance of hypoxia-induced mitochondrial complex III ROS production has been proposed for pulmonary artery smooth muscle cells (PASMCs) (Waypa et al., 2013). Deletion of the Rieske iron-sulphur protein of Complex III from isolated PASMCs abolished mitochondrial ROS generation and increases in [Ca^2+^]_i_ during hypoxia. A similar depletion of hypoxic sensitivity was observed in PASMCs isolated from mice expressing a tunicate alternative oxidase, which allowed for electron flux to continue during concomitant inhibition of mitochondrial complexes III and IV (Sommers et al., 2020). It will be interesting to investigate whether or not complex III ROS have a parallel function in mediating CB type I cell activation in hypoxia. A summary of the estimated importance of succinate metabolism and mitochondrial ROS production in mediating chemoafferent and ventilatory responses to hypoxia is shown in Figure 2.

Whilst mitochondrial IMS ROS are suggested to increase in hypoxia, there is also a significant rise in NAD(P)H (Duchen and Biscoe, 1992; Bernardini et al., 2020). The rise in NAD(P)H occurs over a PO_2_ range approximately equal to that which is known to stimulate the type I cell (Buckler and Turner, 2013) and is dependent on mitochondrial complex I activity (Arias-Mayenco et al., 2018). An elevation in local mitochondrial NADH would likely trigger an increase in cytosolic NADH through action of the malate/aspartate shuttle. A number of reports have indicated that pyridine nucleotides are capable of modifying voltage-gated K^+^ channel activation/inactivation kinetics in primary cells and expression systems (Tipparaju et al., 2005; Kilfoil et al., 2019). In the CB type I cell it has been observed that addition of NADH to the patch pipette prevents hypoxic inhibition of K_v_ currents (Fernandez-Aguera et al., 2015). The identity of specific channels that are regulated by NADH, its functional importance and the mechanism of NADH channel modulation are areas that need to be addressed in the future. However, this data does indicate that NADH may have an important signalling role in the CB during hypoxia.

NAD(P)H, FADH_2_ and FMNH_2_ are key sources of electrons for both mitochondrial and cytosolic redox reactions. Thus, their relative concentration in comparison with their oxidised forms (NAD+, FAD and FMN) can be used to indicate the overall mitochondrial and/or cellular redox status (Bernardini et al., 2020). In the intact CB preparation, Bernardini and colleagues have shown that flavoprotein (Fp) fluorescence decreases rapidly during hypoxia and is accompanied by a concurrent rise in NAD(P)H (Bernardini et al., 2020). As Fp fluorescence represents the oxidised forms of flavoproteins, this data is consistent with an increase in FADH_2_ and FMNH_2_ groups and an overall switch to a more reduced cellular redox status. The authors suggest that such a redox shift leads to stimulation of the redox-sensitive protein PIN1 (located at the cell membrane) which in turn promotes NADPH oxidase activity. Accordingly, pharmacological inhibition of PIN1 was found to partially inhibit membrane depolarisation caused by hypoxia. However, mice lacking the *Gp91 phox* gene exhibit full CB and ventilatory sensitivity to hypoxia (Roy et al., 2000). Thus, there may be alternative signalling pathways subject to changes in the cellular redox status that are still to be revealed. Investigating any direct action of NAD(P)H on ion channel function in the CB could be a logical next step.

## Is a Fall in MgATP Coupled to CB Excitation in Hypoxia?

A fall in mitochondrial activity is likely to modify the adenine nucleotide concentrations within the type I cell and this itself has been suggested to link either directly or indirectly to cellular excitation. In most mammalian cells the majority of ATP is complexed with Mg^2+^, forming MgATP (Gout et al., 2014). Many early studies aimed to measure whole CB adenine nucleotide concentration, including ATP, before and after hypoxia and following addition of mitochondrial poisons. The results were somewhat variable in relation to the effect of hypoxia, with reports of a slight fall in ATP (approximately 20%) by some groups (Obeso et al., 1985), but no significant change observed by others (Acker and Starlinger, 1984; Verna et al., 1990). These data perhaps indicated that a fall in ATP and/or an increase in AMP were unlikely to be important signalling molecules in the type I cell. That said, studies using ATP depleting agents such as 2-deoxy-D-glucose and cyanide have reported a clear negative correlation between ATP content and neurosecretion and/or chemoafferent activity (Obeso et al., 1985; Obeso et al., 1986). All of these experiments measured total ATP content of the CB, which is likely to include contributions from type II cells, neurons, blood vessels and interstitial fluid, in addition to type I cells. ATP is also an important neurotransmitter released by type I cells during hypoxia (Zhang et al., 2000), and this itself can undergo further degradation into ADP, AMP and adenosine by ectonucleotidases including NTPDase1,2,3 and CD73 (Conde and Monteiro, 2004; Salman et al., 2017; Holmes et al., 2018b). Therefore, the total ATP content measured would be dependent on a combination of metabolically derived ATP, the level of stored and released ATP as a neurotransmitter and the rate of extracellular ATP degradation. As such, it is difficult to say, with confidence, that total CB ATP content is an accurate reflection of type I cell MgATP concentration.

To date, no study has been able to make direct measurements of type I cell ATP or MgATP. However, using Mag-indo-1, a Mg^2+^ sensitive fluorescent probe, Varas and colleagues were able to demonstrate that free Mg^2+^ within isolated rat type I cells significantly increased upon exposure to hypoxia (Varas et al., 2007). They concluded that this was a direct consequence of a fall in ATP production and a decrease in MgATP. Similar rises in Mg^2+^ were observed upon exposure to mitochondrial poisons including CN^−^ and oligomycin (a H^+^-ATP synthase inhibitor). It does therefore appear that MgATP falls in the rat type I cell upon exposure to hypoxia. However, it is not apparent if this takes place over the same physiological PO_2_ range known to stimulate the type I cell, and further studies are needed to validate results in other species including mouse and human.

Numerous investigations have reported that oligomycin is capable of activating many aspects of the CB chemotransduction cascade including inhibition of K^+^ channels, rises in [Ca^2+^]_i_ and chemoafferent excitation (Mulligan et al., 1981; Wyatt and Buckler, 2004). This strongly supports the idea that a fall in mitochondrial ATP production is sufficient to cause rapid CB stimulation. Chemoafferent excitation evoked by hypoxia is also severely suppressed following sustained oligomycin application (Mulligan et al., 1981; Shirahata et al., 1987). This is perhaps not completely surprising as some mitochondrial ATP production is likely required to support the increase in type I cell and chemoafferent activity during hypoxia (Bardsley et al., 2021).

It is important to consider precisely how a fall in MgATP may couple to type I cell depolarisation. TASK channels are highly expressed in the CB type I cell and are thought to play a major role in evoking type I cell depolarisation in hypoxia (Buckler et al., 2000; Kim et al., 2009; Mkrtchian et al., 2012; Zhou et al., 2016). MgATP can directly activate TASK-like channels in the type I cell with an EC_50_ of approximately 2.3 mM and saturating concentration of just above 10 mM, both of which are within an estimated physiological range (Williams and Buckler, 2004; Varas et al., 2007). In contrast, substances including free ATP, ADP, AMP and NADH do not appear to directly modulate TASK-like channel activity. Cell attached patches of CB type I cells demonstrate a high degree of single K^+^ channel activity at a membrane potential of −70mV, largely attributable to these TASK-like channels (Varas et al., 2007; Zhou et al., 2016). When these patches are excised into the inside-out configuration, channel activity rapidly decreases to a level of about 10% of control. Interestingly, this run-down can be partially alleviated by 5 mM MgATP, with approximately 50% of channel activity being preserved (Varas et al., 2007). These data suggest that in normoxic physiological conditions, intracellular MgATP acts to maintain the high level of TASK-like channel activity. In hypoxia, the depletion in MgATP would lead to a fall in activity, sufficient to cause cellular depolarisation. However, these data also imply that there are other important intracellular factors, in addition to MgATP, that contribute to TASK channel opening in normoxia, which may be reduced under hypoxic conditions. Such substances are still to be identified and warrant further consideration. It is also not clear precisely how MgATP regulates TASK channels in the type I cell, since they are not known to contain a specific ATP binding pocket and/or be intrinsically sensitive to nucleotides. Thus, there could be a type I cell specific adapter protein, coupled to TASK channels which confers sensitivity to MgATP. Aiming to reveal the identity of this type of protein could be an interesting area for future research.

Though important, TASK channels are unlikely to be the only ion channels involved in CB O_2_ sensing. In TASK1/3 deficient murine CB type I cells, Ca^2+^ elevations and augmented catecholamine release in response to hypoxia are still strongly preserved (Ortega-Saenz et al., 2010; Turner and Buckler, 2013). In rat type I cells, an array of different TASK channel inhibitors all produced significant chemostimulation, but in each case this was well below that elicited by severe hypoxia (O’donohoe et al., 2018). Other potential K^+^ channel candidates that could be regulated by adenine nucleotides include TREK-1 and K_ATP_. Similar to TASK, TREK-1 is known to be activated by physiological intracellular concentrations of MgATP (Enyeart et al., 2002). It has been observed that TREK-1 is expressed in the rat type I cell (Yamamoto and Taniguchi, 2006) but preliminary findings suggest an absence in the human CB (Mkrtchian et al., 2012). Thus, its relevance in CB O_2_ sensing still needs to be defined. An ATP- and glibenclamide-sensitive K^+^ channel has been identified in the type I cell, but it was shown to be inhibited by ATP, therefore making it an unlikely candidate to be responsible for coupling a fall in mitochondrial ATP production with cellular depolarisation in hypoxia (Kim et al., 2011).

An alternative mechanism to cause type I cell depolarisation could be through activation of inward Na^+^, Ca^2+^ or Mg^2+^ currents, carried through TRP channels (Kumar et al., 2006; Kim et al., 2015). Multiple classes of TRP channels have been reported in the type I cell and/or the chemoafferent fibres including TRPV, TRPC and TRPM (Buniel et al., 2003; Roy et al., 2012; Jendzjowsky et al., 2018; Shin et al., 2019). TRPM7 is suggested to be the most highly expressed TRP channel within the mouse CB type I cell, indicative of functional importance (Zhou et al., 2016). TRPM7 has been linked to chemoexcitation upon exposure to leptin, either given exogenously or when elevated during obesity (Shin et al., 2019; Shin et al., 2021). However, its significance in mediating CB type I cell stimulation in hypoxia has not been studied to date. TRPM7 channel activity can be modified by numerous intracellular factors, including the Mg^2+^-complexed nucleotides, MgATP and MgGTP (Nadler et al., 2001; Demeuse et al., 2006). In expression systems, it has been reported that TRPM7 activity significantly increases when MgATP drops below a threshold of approximately 1–2 mM (Nadler et al., 2001). Therefore, it is possible that TRPM7 could be activated in the CB type I cell during hypoxia, in response to a fall in MgATP, and this clearly warrants further investigation.

A number of observations counter the idea that MgATP depletion is necessary to activate the CB during hypoxia. Type I cell Ca^2+^ responses to rotenone alone and rotenone plus hypoxia are non-additive (Ortega-Saenz et al., 2003; Arias-Mayenco et al., 2018). Both of these stimuli would decrease MgATP and, potentially, to a greater extent when applied together. Thus, it would appear that any greater fall in MgATP does not lead to any further type I cell stimulation. However, there is also the possibility that saturating concentrations of rotenone may be sufficient to prevent any further fall in MgATP caused by hypoxia. Direct measurements of MgATP are needed for clarification. The finding that hypoxic responses are robustly maintained in murine type I cells deficient in ATP sensitive TASK1/3 channels also points towards the presence of a non-ATP sensitive mechanism (Ortega-Saenz et al., 2010). Finally, it has been observed that inhibition of voltage-dependent K^+^ current by hypoxia is still apparent in type I cells dialyzed with 3–5 mM MgATP (Lopez-Lopez et al., 1989; Perez-Garcia et al., 2004). Again, this does indicate that there is an O_2_ sensitive signalling mechanism in the type I cell that is independent of MgATP. Evaluation of [Ca^2+^]_i_ and neurosecretory responses to hypoxia measured under conditions of preserved/clamped intracellular MgATP may provide further insight into this matter.

In addition to directly impacting on ion channels, variations in adenine nucleotide concentrations could signal through an intermediate protein/pathway which in turn couples to the cell membrane. A likely candidate would be the AMP-activated protein kinase (AMPK), whose phosphorylation (and thus activation) status is enhanced by an increase in the AMP/ATP ratio. Robust expression of AMPK subunits have been reported in both rodent type I cells (Wyatt et al., 2007; Zhou et al., 2016) and human whole CBs (Mkrtchian et al., 2012). Initial experiments using AICAR, an AMP-mimetic that activates AMPK, were very promising. In rat CBs, AICAR was observed to inhibit background Ba^2+^ sensitive K^+^ currents, cause a rise in [Ca^2+^]_i_ and generate an increase in chemoafferent nerve activity within minutes of drug application (Evans et al., 2005; Wyatt et al., 2007). Compound C, an inhibitor of AMPK, decreased both the type I cell and sensory nerve response to hypoxia. However, these results have been countered by experiments where both AICAR and A769662 (a more direct AMPK activator) were without effect on type I cell TASK channel currents and [Ca^2+^]_i_ (Kim et al., 2014). Furthermore, it has now been suggested that a CB with conditional deletion of both α1 and α2 subunits of AMPK still retains full chemoafferent sensitivity to hypoxia (Mahmoud et al., 2016). This finding was reported from a single experiment and a more complete dataset is still necessary to draw firm conclusions. There is also the possibility that an elevation in alternative O_2_ sensing pathways may have countered the absence of AMPK signalling. However, on current evidence, it does appear that AMPK is not required to activate the CB in hypoxia. Potential roles for AMPK in mediating CB plasticity in cardiorespiratory pathology remain to be defined.

## Is an Elevation in Lactate and Stimulation of Olfactory Receptor 78 (OLFR78) Necessary to Achieve Full CB Chemostimulation in Hypoxia?

When oxidative phosphorylation becomes restricted during low O_2_ conditions, ATP can be partially preserved through an increase in glycolytic flux. Glycolysis is highly sensitive to the ATP/AMP ratio and is maximally stimulated by oligomycin (an ATP-synthase inhibitor) (Tanner et al., 2018; Yagi et al., 2021). To maintain high levels of glycolysis, generated pyruvate is rapidly converted to lactate by lactate dehydrogenase (LDH) (Rogatzki et al., 2015). Exaggerated glucose uptake and lactate removal are also necessary to sustain augmented glycolytic rates (Tanner et al., 2018).

In the CB, it has been hypothesized that an elevation in lactate provides the link between mitochondrial run-down and cellular stimulation during hypoxia (Chang et al., 2015). In support of this idea, it has been reported that rates of glucose uptake in the CB are enhanced during hypoxia (Obeso et al., 1993). Indeed, multiple groups have observed that lactate can also directly stimulate the CB in a concentration dependent manner, with a threshold of approximately 5–10 mM and maximum response achieved at around 30 mM (Chang et al., 2015; Peng et al., 2020; Torres-Torrelo et al., 2021). Arterial blood lactate concentrations can rise to approximately 6–7 mM during exposure to hypoxia (10% inspired) in mice and it is debatable whether this would be sufficient to excite the CB (Chang et al., 2015; Torres-Torrelo et al., 2021). It is, however, possible that local extracellular concentrations of lactate within the CB may be much higher, especially if type I cells have a much higher rate of glycolysis and lactate excretion. Experiments measuring tissue concentrations of lactate in the CB during exposure to hypoxia will be essential to clarify this. Some reports have demonstrated that the peak excitation produced by lactate is similar to or greater than that induced by hypoxia (Chang et al., 2015; Torres-Torrelo et al., 2021). However, others suggest that responses to lactate, even at 30 mM, are relatively modest, measuring only around 30% of that induced by hypoxia (Peng et al., 2020). The difference is likely a consequence of the severity of the hypoxic stimulus intensity used between the different studies, although reduced accessibility to type I cells due to diffusion limitations in the whole CB preparations may also be a contributing factor. It has also been observed that some isolated rat type I cells are unresponsive to 20 mM lactate, as evidenced by no change in membrane potential or outward K^+^ currents (Spiller et al., 2021). The apparent lack of a response could be due to loss of intracellular signalling as experiments were performed in the whole cell patch clamp configuration. To counter this, in the same study, administration of lactate to the *in-situ* CB preparation via the perfusate failed to evoke any chemoafferent excitation at 5 and 20 mM: a situation where lactate would have sufficient access to the tissue and intracellular signalling would be fully preserved. Alternatively, it could be that lactate only excites a proportion of type I cells, but not all. The idea that different type I cells can respond to some but not all stimuli need further exploration. In our own experiments, we have detected that inhibition of glycolysis using iodoacetate leads to rapid and sustained chemoafferent excitation (Holmes et al., 2014). This indicates that there exists a signalling pathway in the CB that can cause chemoafferent excitation independent of an increase in lactate production. Similar measurements have been reported using other inhibitors of glycolysis including 2-deoxy-D-glucose (Obeso et al., 1986). We have also seen that lactate (5 mM) can decrease the elevated chemoafferent activity caused by prolonged glucose deprivation, probably by helping to preserve ATP (Holmes et al., 2014). Thus, under different experimental conditions it has been shown that lactate can stimulate, inhibit, or have no effect on CB function. Its specific role in mediating CB hypoxic sensitivity needs further clarification.

It was originally proposed by Chang and colleagues that OLFR78 was necessary for lactate to activate the CB in hypoxia (Chang et al., 2015). They revealed that *Olfr78* deficient mice failed to display any significant hypoxic ventilatory response. CBs isolated from these mice were also unresponsive to hypoxia and high concentrations of exogenous lactate (30 mM). Another group has reported contradictory results, whereby single CB type I cells isolated from *Olfr78* ^*−/−*^ mice produced neurosecretory responses to hypoxia that were indistinguishable from WT littermates (Torres-Torrelo et al., 2018). These mice also displayed a full hypoxic ventilatory response. The discrepancies between the two groups are hard to reconcile but could be dependent on genetic drift, redundancy mechanisms, animal husbandry and/or background strain.

A third group have recently suggested that *Olfr78* ^*−/−*^ mice have partially attenuated CB responses to mild but not severe hypoxia (Peng et al., 2020). In *ex vivo* experiments, when the hypoxic stimulus was mild, a significant chemoafferent response was still observed, measuring approximately 40% of WT controls. In whole animal breathing experiments, ventilation in response to 12% O_2_ was slightly reduced in *Olfr78* ^*−/−*^ mice when normalized to O_2_ consumption. There was also a considerable fall in P_a_CO_2_ consistent with hyperventilation. Interestingly, this group also showed that CBs from *Olfr78* KO mice were still sensitive to lactate. Thus, they concluded that even though OLFR78 played a role in CB O_2_ sensing, its activation in mild hypoxia must be dependent on a substance other than lactate. OLFR78 can respond to many substances including the short chain fatty acids acetate, propionate and butyrate, but so far stimulation of the CB by these substances has only been observed at supraphysiological concentrations (Peng et al., 2020). Identification of a substance that can stimulate OLFR78 in the CB at physiological concentrations could be of considerable importance, especially given that *Olfr78* is one of the most highly expressed genes in the CB (Chang et al., 2015).

For lactate that is produced intracellularly to activate OLFR78 it is assumed that it must first leave the cell to gain access to the extracellular facing binding site. It has been shown that monocarboxylate transporters 2 and 4 (MCT-2 and MCT-4) are expressed in type I cells, but not MCT-1, providing potential route for lactate both into and out of the cells (Torres-Torrelo et al., 2021). In isolated type I cell preparations, the small volume recording chamber is constantly renewed by the external perfusate, thereby preventing any extracellular lactate accumulation. Yet, many studies have demonstrated that isolated type I cells retain intrinsic O_2_ sensitivity and are capable of eliciting rises in mitoROS, increases in [Ca^2+^]_i_ and neurosecretion (Fernandez-Aguera et al., 2015; Arias-Mayenco et al., 2018). This counters the idea of autocrine/paracrine lactate signalling being essential to stimulate the type I cells in hypoxia.

Collectively, these data do not rule out lactate being involved in CB O_2_ sensing but do suggest that it most likely does not act through OLFR78. If lactate does reach high mM concentrations in CB tissue during hypoxia, then it could act by alternative mechanisms such as causing intracellular acidification (Torres-Torrelo et al., 2021). What is apparent is that a very large rise in lactate concentration would be required to stimulate the CB. This would likely only occur when falls in MgATP are very severe, possibly limiting its role to the terminal hypoxia section of the chemoafferent PO_2_-response curve.

## Conclusion-Putting Together the Full Response to Hypoxia

**FIGURE 3:**
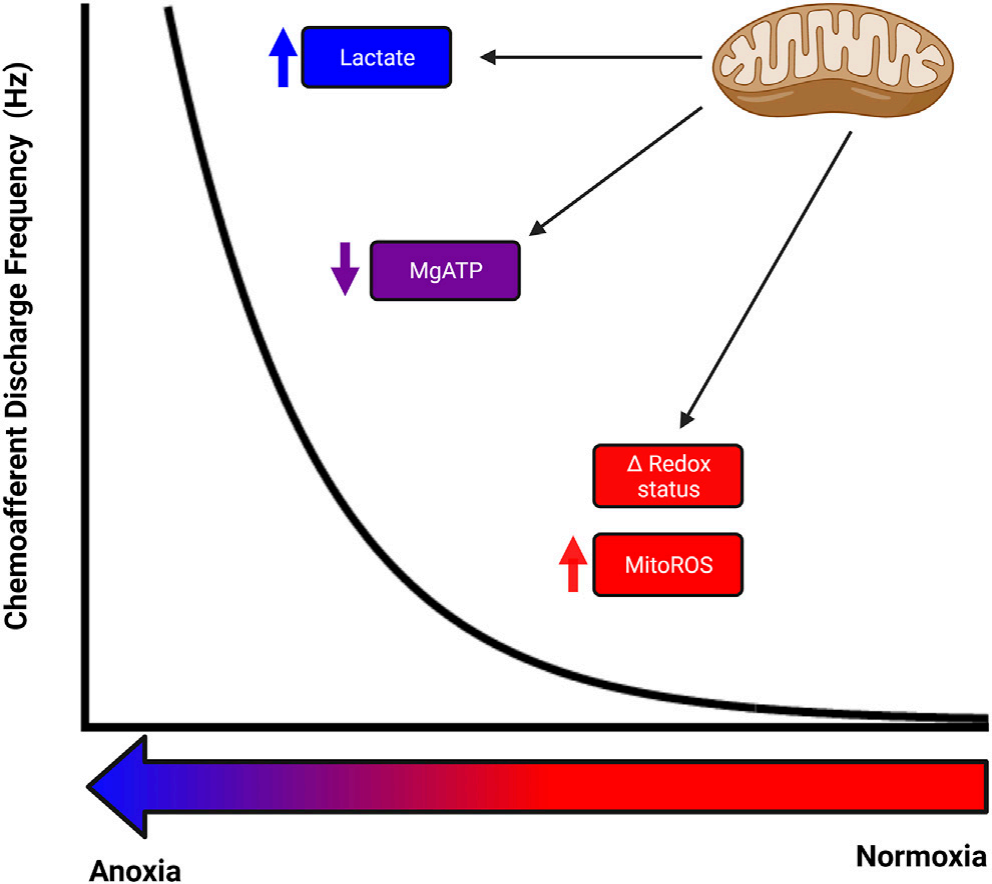
Potential for numerous mitochondrial related signalling mechanisms to co-operate to produce the full carotid body response to hypoxia. Different mitochondrial related signalling mechanisms could be responsible for the rise in chemoafferent discharge at different levels of hypoxia, with an alteration in cellular redox status and mitochondrial reactive oxygen species acting during mild-moderate hypoxia, a decrease in MgATP being important during moderate-severe hypoxia and an increase in lactate contributing during severe hypoxia. There may well be significant overlap between these signalling pathways and potential for modification/adaptation.

Given the non-linear shape of the chemoafferent and ventilatory response curves to O_2_ (Kumar and Prabhakar, 2012), the existence might be construed of a single sensor mechanism with a similarly complex and varying sensitivity across the entire physiological range of PO_2_. Alternatively, a number of sensor mechanisms, each with its own particular, potentially overlapping sensitivity, might act together synergistically to provide the entire response. The conflicting data to date, tends to suggest the latter as the most probable means by which the CB type I cell enables and maintains its exquisite O_2_ sensitivity between the extremes of hyperoxia and anoxia. As suggested by Prabhakar, this may involve multiple O_2_ sensor mechanisms, both mitochondrial and extra-mitochondrial, acting upon multiple neurotransmitter systems within a so-called “chemosome,” to generate both the high and broad-range sensitivity as well as the rapid response that characterizes this receptor system (Prabhakar, 2006). In addition, inhibitory mechanisms within the chemosome could act in a protective manner during periods of prolonged stimulation, such as the chronic hypoxia associated with numerous cardiorespiratory pathologies. We suggest here, that such a chemosome might exist within the distinctive CB type I mitochondria themselves, with perhaps mitoROS and cellular redox signalling contributing more towards the higher PO_2_ portion of the response curve, with a fall in MgATP and/or lactate contributing more towards the severe hypoxia/anoxic portions of the curve (Figure 3).

Mitochondrial signalling may act either independently or in partnership with extra-mitochondrial O_2_-sensors, to generate the full, physiological response to acute hypoxia and perhaps even to the remodelling plasticity observed in the CB and its reflexes with chronic hypoxia. One of the most influential hypotheses of CB hypoxic chemotransduction involves O_2_ dependent generation of H_2_S (Olson et al., 2008; Peng et al., 2010). Compelling evidence has been presented demonstrating that mice deficient in the H_2_S generating enzyme cystathionine γ-lyase (CSE) have an almost completely absent hypoxic ventilatory response (Peng et al., 2010). CBs isolated from these mice have a diminished chemoafferent response to hypoxia whilst retaining normal hypercapnic sensitivity. The rise in H_2_S in hypoxia is thought to be a consequence of reduced CO synthesis subsequent to a decrease in the haem-oxygenase 2 activity (Yuan et al., 2015). Although H_2_S is able to rapidly and reversibly inhibit BK_Ca_ channels (Telezhkin et al., 2010) it is also a classical inhibitor of cytochrome c oxidase. The calculated concentrations of H_2_S required to excite isolated rat type I cells also evoke an increase in NADH, consistent with concurrent mitochondrial inhibition (Buckler, 2011). This could provide a unifying link between these two hypotheses. The endogenous role of H_2_S in modifying the O_2_ sensitivity of the unique CB type I cell mitochondria requires further exploration as does the intriguing possibility of a novel functional role of mitochondrial generated reactive sulphur species (Olson, 2021).

In this review we have discussed some of the key mitochondrial related signalling mechanisms in the CB known to date. It is likely that novel mitochondrial and extra-mitochondrial signalling mechanisms will emerge in the future as we move towards a more complete understanding. Indeed, very recent data suggests that local mitochondrial temperature transients contribute to type I cell membrane depolarisation (Rakoczy et al., 2022). Furthermore, a fail-safe mechanism has been recently proposed based on model testing, whereby low O_2_ leads to disruption of transporter activity involved in maintenance of intracellular pH (Duffin, 2020). The ensuing intracellular acidification is predicted to be sufficient to cause type I activation. The model also indicates that such a mechanism could account for significant CO_2_-O_2_ stimulus interaction. Clearly, experimental data is now required to validate these model data. Given the emerging prominence of the CB hyperactivity in initiating hypertension and cardiac arrhythmia (Iturriaga et al., 2021) future studies are needed to investigate how mitochondrial O_2_ sensitivity and signalling is altered in pathology.
